# Toll-like Receptor 4 Inflammatory Perspective on Doxorubicin-Induced Cardiotoxicity

**DOI:** 10.3390/molecules28114294

**Published:** 2023-05-24

**Authors:** Natticha Sumneang, Pongpan Tanajak, Thura Tun Oo

**Affiliations:** 1 Department of Medical Science, School of Medicine, Walailak University, Nakhon Si Thammarat 80160, Thailand; 2 Research Center in Tropical Pathobiology, Walailak University, Nakhon Si Thammarat 80160, Thailand; 3 Department of Physical Therapy, Rehabilitation Center, Apinop Wetchakam Hospital, Kaeng-Khoi District, Saraburi 18110, Thailand; ptanajak2531@gmail.com; 4 Department of Biomedical Sciences, University of Illinois at Chicago, College of Medicine Rockford, Rockford, IL 61107, USA; drthura@uic.edu

**Keywords:** doxorubicin, Toll-like receptor 4, cardiotoxicity, heart

## Abstract

Doxorubicin (Dox) is one of the most frequently used chemotherapeutic drugs in a variety of cancers, but Dox-induced cardiotoxicity diminishes its therapeutic efficacy. The underlying mechanisms of Dox-induced cardiotoxicity are still not fully understood. More significantly, there are no established therapeutic guidelines for Dox-induced cardiotoxicity. To date, Dox-induced cardiac inflammation is widely considered as one of the underlying mechanisms involved in Dox-induced cardiotoxicity. The Toll-like receptor 4 (TLR4) signaling pathway plays a key role in Dox-induced cardiac inflammation, and growing evidence reports that TLR4-induced cardiac inflammation is strongly linked to Dox-induced cardiotoxicity. In this review, we outline and address all the available evidence demonstrating the involvement of the TLR4 signaling pathway in different models of Dox-induced cardiotoxicity. This review also discusses the effect of the TLR4 signaling pathway on Dox-induced cardiotoxicity. Understanding the role of the TLR4 signaling pathway in Dox-induced cardiac inflammation might be beneficial for developing a potential therapeutic strategy for Dox-induced cardiotoxicity.

## 1. Introduction

Cancer is a major public health problem worldwide. Cancer is a complicated disease caused by internal factors (e.g., inherited mutations, hormones, and immune conditions) and environmental factors (e.g., diet, radiation, and infectious organisms) [1,2]. According to a report from the World Health Organization, cancer is one of the leading causes of death worldwide [3]. In the United States of America, there were approximately 1.98 million new cases of diagnosed cancer in 2022 [4]. Furthermore, the number of cancer cases around the world is expected to reach around 26 million by 2030, with 17 million deaths per year [5].

Currently, doxorubicin (Dox) is one of the most used chemotherapeutic drugs for various types of cancer, such as breast, lung, ovarian, and thyroid cancers [6,7]. Although it is able to combat tumor cells, it is also harmful to normal cells, including cardiomyocytes [7,8,9]. In addition, several studies have reported the potential mechanisms of Dox-induced cardiotoxicity mediated by mitochondrial dysfunction, DNA damage, oxidative stress, and apoptosis [2,7,9]. A growing body of research suggests that aseptic inflammation might also play a plausible role in Dox-induced cardiotoxicity due to the activation of the innate immune system after Dox treatment [10,11,12]. A number of studies showed that Dox treatment upregulates the expressions of pro-inflammatory cytokines in cardiac tissue, such as tumor nuclear factor (TNF)-α, interleukin (IL)-1β, and IL-6, via activation of the nuclear factor-κB (NF-κB), triggering the progression of cardiovascular diseases and other adverse cardiac events [13,14,15]. Dox-induced cardiotoxicity mainly limits the cumulative dose of Dox in clinical settings. Cardiotoxicity is a potentially lethal condition and also a well-known adverse effect of Dox; however, the underlying molecular mechanism of Dox-induced cardiotoxicity, particularly related to inflammation, is not fully understood. Although the underlying mechanisms of Dox-induced cardiotoxicity remain complex, the role of cardiac inflammation in Dox-induced cardiotoxicity has become a focus for researchers in recent years.

Toll-like receptor 4 (TLR4), an important member of the TLR family, is part of the innate immune system that responds to the endogenous and exogenous signals and triggers pathophysiological functions in organs, including the heart [16,17,18,19]. The precise molecular mechanisms of TLR4 signaling have been elucidated. Upon binding to a specific ligand with the help of myeloid differentiation factor 2 (MD2), the TLR4 signaling pathway is activated, followed by recruiting the intracellular adaptor molecules and releasing the inflammatory mediators [15,20,21]. Interestingly, there is increasing evidence that Dox-induced TLR4 signaling pathway activation is implicated in cardiac adverse effects, which manifest as left ventricle (LV) impairment [20,22,23]. In support of this evidence, a previous study demonstrated that Dox-induced cardiac adverse effects were completely alleviated in TLR4 knockout mice [22]. Therefore, TLR4 signaling is thought to be involved in the mechanism contributing to Dox-induced cardiotoxicity, and inhibition of TLR4 is considered to be one the potential interventions against Dox-induced cardiomyopathy [22,23].

The aim of this review is to comprehensively summarize and discuss in vitro, in vivo, and clinical reports on the plausible mechanism of TLR4 on Dox-induced cardiotoxicity, including inflammatory mediators, oxidative stress, and apoptosis in cardiomyocytes, as well as cardiac remodeling and cardiac function. To support the upcoming clinical trials that will help to either prevent or lessen Dox-induced cardiotoxicity, we also aim to thoroughly compile the currently available evidence of TLR4 inhibition.

## 2. The Mechanism of TLR4 on Dox-Induced Cardiotoxicity

Growing evidence has shown that Dox activates the innate immune system, which is one of the expected components of the response against tumor cells [2,9]. However, Dox-induced innate immune activation provokes the release of inflammatory cytokines in a number of tissues, including the heart, intestine, brain, and liver, which results in inflammation in non-targeted organs [9,10,24,25]. To date, several studies have demonstrated that Dox-induced cardiac inflammation is strongly linked to Dox-induced cardiotoxicity [10,14,26,27]. Dox induces NF-κB activity to promote cardiac inflammation by upregulating TNF-α, IL-1β, and IL-6 expression [15,26].

TLR4 is responsible for the innate immune response; thus, the activation of TLR4 has become one of the most attractive targets in recent years [28,29]. Moreover, the expression of TLR4 is also found in the cardiomyocytes in addition to the immune cells [21]. This receptor responds to exogenous and endogenous ligands: pathogen-associated molecular patterns (PAMPs) and damage-associated molecular patterns (DAMPs) [28]. TLR4 recognizes the bacterial LPS as a major PAMP with the help of its co-receptor MD2 [21]. In addition, the TLR4 signaling pathway can also be activated by various endogenous DAMPs, namely alarmin protein, including high-mobility group protein box 1 (HMGB1) and the heat shock protein family (Hsps) [28,30]. Upon ligand binding, TLR4 dimerization occurs, followed by activation of the myeloid differentiation primary response protein 88 (MyD88); the downstream signaling pathways propagate NF-κB phosphorylation via reducing the inhibitory κB kinase (IKK) response, leading to upregulation of inflammatory cytokines and cardiac inflammation [21]. In addition to NF-κB activity, components of mitogen-activated protein kinases (MAPKs), including extracellular signal-regulated kinase 1 and 2 (ERK1/2), c-Jun N-terminal kinase (JNK), and p38, are also activated as downstream signal transducers of MyD88 to contribute to the regulation of pro-inflammatory responses [18,21].

**Figure 1:**
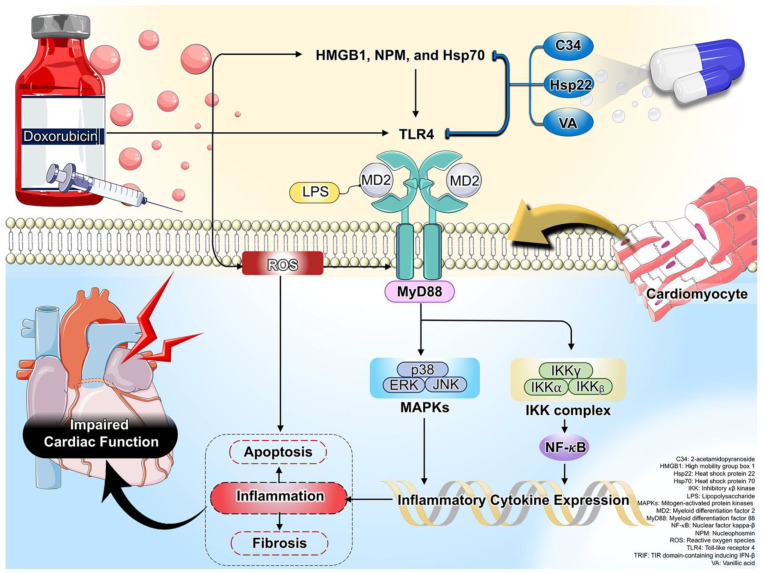
A schematic presentation of oxidative stress and inflammation-related mechanisms via TLR4 in Dox-induced cardiotoxicity. Activation of TLR4 was exhibited by HMGB1, NPM, and Hsp70, leading to increased cardiac inflammation, apoptosis, and fibrosis, and impaired cardiac function in Dox treatment. Its subsequent detrimental effects were effectively attenuated by treatment with C34, Hsp22, and VA.

To date, a growing body of evidence demonstrates that Dox induces the release of PAMP and DAMP, resulting in TLR4-mediated cardiac inflammation that contributes significantly to cardiotoxicity [10,15,28,31]. Based on evidence from in vitro and in vivo studies, we review and discuss how Dox causes cardiac TLR4 upregulation and inflammation in this review. A simplified overview of the mechanistic activation of TLR4 in Dox-induced cardiotoxicity is shown in Figure 1. Therefore, inhibition of TLR4 would have a therapeutic benefit against Dox-induced cardiotoxicity.

## 3. The Effects of Dox on TLR4 Expression in Cardiomyocytes: Reports from In Vitro Studies

To date, the relationship between the role of TLR4 and Dox administration is still scarce; however, increasing evidence from in vitro studies suggests that Dox-mediated cardiotoxicity is related to the upregulation of cardiomyocyte TLR4 expressions [32,33,34]. Li et al. reported that Dox administration upregulated the expression of TLR4 in human cardiomyocyte cell lines [32]. In addition, Feng et al. demonstrated that Dox treatment significantly enhanced the TLR4 signaling pathway in H9c2 cardiomyocytes, as evidenced by increased TLR4 expression and its downstream signaling pathways, including MyD88, NF-κB, IL-1 β, IL-6, and TNF-α, along with decreased IkBα expression [27]. According to these two in vitro results, we speculate that Dox administration triggered cardiac inflammation via the TLR4-MyD88 signaling pathway. However, further research is necessary to fully understand the underlying molecular signaling of Dox-induced TLR4 signaling pathway activation in cardiomyocytes.

Traditionally, TLR4 ligands, PAMPs, and DAMPs bind to MD2 to activate the TLR4 signaling pathway in cardiomyocytes, resulting in cardiac inflammation [18,35]. The DNA-binding nuclear protein HMGB1, which regulates gene transcription and nucleosome stability, has a variety of biological functions in clinicopathological conditions [36]. It is considered a cytokine involved in the activation of innate immunity after being actively released from cells or passively released upon cell injury [36]. Interestingly, TLR4 functions as the major HMGB1 receptor [37]. Disulfide HMGB1 activates the TLR4 complex, binding to MD2, which triggers dimerization of TLR4 that can stimulate downstream signal transduction molecules (e.g., NF-κB) to produce pro-inflammatory cytokines [37]. A previous study demonstrated that the release of HMGB1 from the cells was increased following Dox treatment [38]. Moreover, the oxidative stress and DAMPs were considered to be major stimulators of HMGB1 release, activating inflammation through the TLR4 signaling pathway [38]. Therefore, Dox is closely related to altered HMGB1 levels, leading to induced cardiotoxicity.

In addition to HMGB1, there is also nucleophosmin (NPM), which behaves similarly to an alarmin protein released in response to cellular excess due to severe injury [39]. Similar to HMGB1, NPM can also bind to the TLR4 signaling cascade, leading it to exerting pro-inflammatory cytokines [39]. Interestingly, a previous study showed that Dox induced nucleolar stress, subsequently disrupting and releasing the NPM from the nucleolus. Then, the extracellular NPM induced inflammation via TLR4 signaling pathway activation [39]. To date, there is only one study that has shown that NPM can bind to the TLR4 signaling cascade, promoting pro-inflammatory function following the Dox treatment of human cardiac mesenchymal progenitor cells (hCmPCs) [39]. Thus, NPM is a novel ligand of TLR4 that activates inflammation in Dox-treated cardiotoxicity [39].

Inhibition of TLR4 via genetic deletion suppressed Dox-induced cardiotoxicity [27,31,32]. This evidence was provided by previous studies, showing that genetic ablation of TLR4 not only reduced inflammation but also decreased apoptosis in cardiomyocytes after exposure to Dox [27,31,32]. Although molecular signaling through TLR4 has not been demonstrated in the genetic deletion of TLR4 on apoptosis in cardiomyocytes after Dox exposure, the aforementioned discussions suggest that pro-inflammatory cytokines [27], mediated by TLR4, were suppressed, resulting in a decrease in cardiomyocyte apoptosis, as evidenced by the decrease in apoptotic proteins (Bax and cleaved caspase-3) and the increase in anti-apoptotic proteins (Bcl-2) [31,32]. These in vitro studies demonstrate the plausible involvement of TLR4 in Dox-induced cardiotoxicity via the implication in cardiomyocyte apoptosis.

C34, a potent and selective antagonist of TLR4, reduced NPM secretion in Dox-treated hCmPcs, further confirming that TLR4 regulated the secretion of NPM via autoregulatory feedback between TLR4 and NPM following Dox treatment [39]. Therefore, these findings revealed that inhibition of TLR4 might be a novel therapeutic strategy in reducing Dox-induced cardiotoxicity. Reports regarding the effects of Dox on TLR4 expression in cardiomyocytes in in vitro studies are summarized in Table 1.

## 4. The TLR4 Expression in Dox-Induced in Cardiomyocytes: Reports from In Vivo Studies

Several studies have demonstrated that Dox treatment leads to impaired cardiac function in rodents, as assessed via echocardiography and invasive hemodynamic assessment [14,23,40]. Cellular and molecular studies have also been carried out using the cardiomyocytes isolated from these Dox-treated animals, and the results are largely consistent with the findings from the in vitro models discussed in the previous section. Previous in vivo studies have shown that Dox enhanced the expression of TLR4 in the cardiac tissue of rodents [14,23,40]. Furthermore, the levels of TLR4 ligands, such as HMGB1 and Hsp70, were also significantly increased in the cardiac tissue of Dox-treated mice [40]. This result further confirms that the TLR4 signaling pathway was activated following Dox treatment, leading to the triggering of NF-κB activity, which leads to the generation of pro-inflammatory cytokines in cardiac tissue, as evidenced by increases in TNF-α, IL-6, IL-13, monocyte chemotactic protein (MCP-1), and transforming growth factor (TGF)-β1 [14,40]. Consistently, the expression of cardiac macrophage markers, including CD45 and CD68, was also elevated in Dox-treated mice [14]. Taken together, elevated cardiac macrophages expressing TLR4 can lead to overwhelming pro-inflammatory cytokines in Dox-treated animals [14,40]. In addition to cardiac inflammation, cardiac remodeling was also observed in Dox-treated mice [40], which was attributed to the fact that TGF-β1 was increased in the hearts of these mice [40]. TGF-β1 is involved in the cardiac remodeling process, since it is a multifunctional cytokine and a growth factor that plays multiple roles in inflammation and fibrosis [41]. Therefore, an increase in cardiac TGF-β1 expression promoted collagen accumulation in the heart of Dox-treated mice, as indicated by increasing α-smooth muscle actin (α-SMA) [40].

Dox induces ROS through redox reactions due to its quinone component, with the resulting production of the superoxide anion (O_2_^−^), hydrogen peroxide (H_2_O_2_), and hydroxyl radical (∙OH) [8]. This ROS can lead to lipid peroxidation, as indicated by an increase in malondialdehyde (MDA) that was observed in the hearts of Dox-treated rats [14]. In addition, this ROS can trigger apoptosis in cardiac tissue in Dox-treated mice, as evidenced by an increase in Bax, cytochrome *c*, and TUNEL^+^ cells, as well as a decrease in Bcl-2 in cardiac tissue [14]. According to in vitro studies [31,32], an in vivo study has also shown that increasing TLR4 activation was implicated in cardiac apoptosis in Dox-treated mice [14]. This could be due to the oxidative stress associated with the increase in TLR4 expression that further promotes inflammation [42], which, in turn, contributes to apoptosis. Therefore, oxidative stress not only directly induces cardiac apoptosis per se but also promotes inflammation via increasing TLR4 expression, leading to cardiac apoptosis in Dox-treated mice.

Therefore, in vivo studies have suggested that enhanced activation of the TLR4 signaling pathway is involved in Dox-induced cardiotoxicity, which includes cardiac inflammation, remodeling, oxidative stress, and apoptosis via activation of TLR4, resulting in impaired cardiac function [14,23,40]. The in vivo evidence pertinent to TLR4 expression in Dox-induced cardiotoxicity is summarized in Table 2.

## 5. The Potential Role of TLR4 Inhibition in Dox-Induced Cardiotoxicity: Reports from In Vivo Studies

The evidence from in vivo studies on the effects of TLR4 inhibition in Dox-induced cardiotoxicity is summarized in Table 3. Since several studies, including both in vitro and in vivo studies, have revealed that activation of the TLR4 signaling pathway is partly involved in Dox-induced cardiotoxicity, targeting the TLR4 pathway has become a possible therapeutic strategy [14,22,23]. In vitro reports have shown that either silencing or knockout TLR4 attenuated Dox-induced cardiotoxicity [27,31,32,39], and in vivo study reports have also demonstrated that genetic ablation of TLR4 improved cardiac function in Dox-treated mice, as indicated by increased LV end-systolic pressure–volume relation (LVESPVR), cardiac output (CO), and stroke volume (SV) [22,31]. In addition, the molecular levels, including inflammatory markers, fibrosis accumulation, oxidative stress, and apoptotic markers, in cardiac tissue of Dox treatment were alleviated in TLR4-deficient mice [22,31] (Table 3).

In addition to genetic ablation of TLR4 models in cardiomyocytes, the blockade of TLR4 by using the pharmacological compound vanillic acid (VA), a TLR4 antibody, and adeno-associated virus–heat shock protein 22 (AAV-Hsp22) injection has also exerted protective effects in Dox-induced cardiotoxicity [14,23]. A previous study has shown that VA, a natural compound of the phenolic acid family, can suppress the TLR4-induced inflammatory response in Dox-treated mice [23]. This study was the first report of the helpful effect of VA via inhibiting TLR4 [23]; however, future studies need to validate which structure of VA blocks TLR4 signaling. In addition, a reduction in TLR4 by VA resulted in decreased cardiac MDA levels and further improved cardiac function in Dox-treated mice [23]. In addition to intervention with a pharmacological agent, Hsp22, a novel TLR4 ligand, has been shown to exert anti-apoptotic and anti-inflammatory effects in other diseases [43,44]. According to this study, adeno-associated virus Hsp22 alleviated cardiac inflammation and apoptosis by blocking TLR4 activation, leading to an improvement in cardiac function in Dox-induced cardiotoxicity in mice [14]. It is proposed that the TLR4-dependent signaling pathway renders Dox-mediated adverse cardiac effects, and the findings implied that inhibitions of TLR4 might attenuate pathophysiological key mechanisms of Dox-induced cardiotoxicity.

As previously discussed, Dox-induced cardiotoxicity can be due to several complex mechanisms. The TLR4-independent signaling pathway, involving oxidative stress, apoptosis, and so on, might play a significant role in Dox-induced cardiotoxicity [8,45]; however, only inhibition of TLR4 could attenuate the adverse effects on the heart after Dox administration [14,22,23,31,32,39]. Therefore, both TLR4-dependent and -independent signaling pathways could be considered as the fundamental mechanisms linked to the Dox-mediated cardiotoxicity, and targeting TLR4 is sufficient to reduce Dox-mediated cardiotoxicity.

Although several studies have shown that targeting TLR4 exerted cardioprotective effects in Dox-treated mice [14,22,23], only one study demonstrated completely contradictory findings [40]. Immunomodulation of TLR4 signaling with a TLR4-neutralizing antibody exacerbated cardiac fibrosis and impaired cardiac function by promoting higher inflammatory gene expression and, subsequently, an increase in inflammatory cytokines [40]. This might be possible due to the isotope of the TLR4 antibody used in that study [40]. Ma et al. injected TLR4 immunoglobulin G (IgG) antibody in Dox-treated mice [40]. Since IgG enhances pro-inflammatory response and stimulates immune cells, this might be the reason why TLR4 antibody administration did not attenuate Dox-induced cardiotoxicity. Furthermore, immunomodulation with TLR4 antibody administration resulted in a disruption of downregulating p38, which eventually suppressed autophagy and led to further cardiac dysfunction in Dox-treated mice [40]. Knockout of the TLR4 gene, a technique used by Raid et al. and Yao et al., showed greater efficacy on Dox-induced cardiotoxicity compared to TLR4 immunomodulation with TLR4 antibodies in Dox-treated mice [22,31]. Moreover, the administration of TLR4 inhibitors with Hsp22 and VA showed a more positive effect than the use of TLR4 antibodies [14,23,40]. Therefore, depletion of TLR4 by either genetic modification or pharmacological agents might be more effective than TLR4-neutralizing antibodies in Dox administration.

**Table 3:** The effects of TLR4 inhibition in Dox-induced cardiotoxicity: reports from in vivo studies.

Model	Protocol (Dose, Duration)	Intervention of TLR4 Inhibition (Agent, Dose, Route, Duration)	Major Findings					Interpretation	Ref.
			Cardiac Function	Inflammatory Markers	Cardiac Remodeling/Fibrosis	Oxidative Stress	Apoptosis		
C57BL/6J mice	Dox (20 mg/kg, i.p., single dose) -Sacrifice on day 5	TLR4^−/−^	↑ LVESPVR	n/a	n/a	n/a	↓ TUNEL^+^	TLR4 deficiency attenuated Dox-induced cardiac apoptosis and dysfunction in mice.	[31]
C57BL/10ScSn mice	Dox (20 mg/kg, i.p., single dose) -Sacrifice on day 5	TLR4^−/−^	↑ SV ↑ CO	↓ TNF-α ↓ CD3+ ↓ CD11b+ ↓ CD8a+	n/a	↓ Lipid peroxidation ↓ Nitrotyrosine	↓ Bax ↑ Bcl-2 ↓ TUNEL^+^	TLR4 deficiency rescued Dox-induced cardiotoxicity in mice.	[22]
C57BL/6J mice	Dox (3.4 mg/kg/wk, i.p., 8 wk) -Sacrifice on day 103	TLR4ab (first dose was 200 µg/mg and following doses were 100 µg/mg, tv.i., on day 64, 68, 72, 79, 86, 96, and 100 after Dox injection)	↓ %LVEF ↓ %LVFS	↔TLR4 ↔ HMGB1 ↔ Hsp70 ↑ MCP-1 ↑ IL-13 ↑ TGF-β1	↑ Fibrosis ↑ α-SMA	n/a	n/a	Immunomodulation of TLR4 exacerbated cardiac dysfunction in Dox-treated mic by increasing inflammation and fibrosis.	[40]
C57BL/6J mice	Dox (15 mg/kg, i.p., single dose) -Sacrifice on day 21	AAV-Hsp22 (5 × 10^10^ viral genome particles, tv.i., single dose, before Dox injection for 4 wk)	↑ %LVEF	↓ TLR4 ↓ TNF-α ↓ IL-6 ↓ NF-kB ↓ CD68 ↓ CD45	n/a	n/a	↓ Bax ↑ Bcl-2 ↓ Cyt *c* ↓ TUNEL^+^	Hsp22 protected the heart against Dox-induced cardiotoxicity via inhibited TLR4/NF-kB signaling pathway in mice.	[14]
Wistar rats	Dox (2.5 mg/kg/3 doses/wk, i.p., 2 wk) -Sacrifice on day 28	VA (10, 20, and 40 mg/kg/d, p.o., 4 wk, before Dox injection for 14 days)	n/a	↓ TLR4	n/a	↓ MDA	n/a	VA protected the heart against Dox-induced cardiotoxicity via inhibited TLR4 signaling pathway in rats.	[23]

n/a: Data are not available; AAV-Hsp22: adeno-associated virus–heat shock protein 22; Bax: Bcl-2-associated X; Bcl-2: B-cell lymphoma-2; CO: cardiac output; Cyt *c*: cytochrome c; Dox: doxorubicin; HMGB1: high-mobility group box 1; Hsp70: heat shock protein 70; i.p.: intraperitoneal injection; IL-13: interleukin 13; IL-6: interleukin-6; LVEF: left ventricular ejection fraction; LVESPVR: left ventricular end-systolic pressure-volume relation; LVFS: left ventricular fractional shortening; MCP-1: monocyte chemotactic protein 1; MDA: malondialdehyde; NF-kB: nuclear factor kappa-light-chain-enhancer of activated B cells; p.o.: per orally; SV: stroke volume; TGF-β1: tumor growth factor β1; TLR4: Toll-like receptor 4; TNF-α: tumor necrosis factor-alpha; TUNEL: terminal deoxynucleotidyl transferase mediated dUTP nick end labeling; tv.i.: tail vein injection; VA: vanillic acid; α-SMA: α-smooth muscle actin; ↑: Increase; ↓: Decrease; ↔: No change.

## 6. The Effects of Dox on Systemic TLR4 Expression: Reports from Clinical Studies

Clinical studies have shown the important role of systemic TLR4 on cardiac function in patients with hematological malignancy who received a Dox regimen [46,47]. After 6 months of Dox administration, blood sampling was collected to determine TLR4 expression, together with measuring the non-invasive cardiac function in Dox-treated patients [46,47]. The results of these two studies were consistently showed that these patients had impaired cardiac function after 6 months of Dox treatment, whereas TLR4 expressions were significantly increased in peripheral blood sampling [46,47]. In addition, a negative correlation between left ventricular ejection fraction and TLR4 expression was observed after 6 months of Dox treatment [47]. These findings were explained by the fact that Dox disrupts the intestinal mucosa integrity, leading to leakage of endotoxin, LPS, into the systemic circulation [48,49,50]. Therefore, the systemic inflammation was stimulated via the interaction of LPS and TLR4 [51,52,53]. This suggested that a decline in cardiac function is associated, in part, with systemic TLR4 expression. Therefore, Dox-induced cardiotoxicity is not only caused by cardiac TLR4 inflammation and cardiac dysfunction but also linked with systemic TLR4 elevation. To date, there is no available clinical evidence regarding the role of TLR4 in Dox-induced cardiotoxicity. In addition, multi-omics integration [54,55] to reveal the mechanism of TLR4 on Dox-induced cardiotoxicity is needed to better understand health and disease, and in certain cases, as part of medical care in the future. A summary of clinical studies is shown in Table 4. In addition, a schematic presentation of oxidative stress and inflammation-related mechanisms via TLR4 in Dox-induced cardiotoxicity is summarized and shown in Figure 1.

## 7. Conclusions

There is an accumulation of evidence from in vitro and in vivo studies and clinical reports demonstrating that the intensive arm of the TLR4 in Dox-induced cardiotoxicity, especially cardiac inflammation, leads to cardiac remodeling and impaired cardiac function. Therefore, the mechanism of understanding the role of the TLR4 signaling pathway in Dox-induced cardiac inflammation might be beneficial for developing a potential therapeutic strategy for Dox-induced cardiotoxicity in the near future.

## Figures and Tables

**Table 1:** The effects of Dox on TLR4 expression in cardiomyocytes: reports from in vitro studies.

Model	Protocol (Drug, Dose, Duration)	Major Findings		Interpretation	Ref.
		Inflammatory Markers	Apoptosis		
AC16 cells	Dox (5 µM, 24 h) si-TLR4	Dox ↑ TLR4 Dox + si-TLR4 ↓ TLR4	Dox ↑ Bax ↑ Caspase-3 ↓ Bcl-2 Dox + si-TLR4 ↓ Bax ↓ Caspase-3 ↑ Bcl-2	Dox treatment increased the expressions of TLR4 and apoptotic proteins in AC16 cells.	[32]
H9c2	Dox (5 µM, 24 h) si-TLR4	Dox ↑ TLR4 ↑ MyD88 ↑ IL-1 ↑ IL-6 ↑ NF-kB ↑ TNF-α ↓ IkBα Dox + si-TLR4 ↓ TLR4 ↓ MyD88 ↓ IL-1 ↓ IL-6 ↓ NF-kB ↓ TNF-α ↑ IkBα	Dox n/a Dox + si-TLR4 n/a	Dox administration induced TLR4 signaling pathway activation in H9c2.	[27]
Neonatal cardiomyocytes	Dox (0.5 µM, 6 h) TLR4^−/−^	Dox ↑ HMGB1 Dox + TLR4^−/−^ n/a	Dox ↑ Caspase-3 Dox + TLR4^−/−^ ↓ Caspase-3	Dox treatment increased the release of HMGB1 and caspase-3 expression in neonatal cardiomyocytes.	[31]
hCmPCs cells	Dox (1 µM, 8 h) C34 (100 µM, 30 min)	Dox ↑ NPM Dox + C34 ↓ NPM	Dox n/a Dox + C34 n/a	Dox treatment increased the level of NPM in hCmPCs cells.	[39]

n/a: Data are not available; AC16: human cardiomyocyte cell line; Bax: Bcl-2-associated X; Bcl-2: B-cell lymphoma-2; C34: 2-acetamidopyranoside; Dox: doxorubicin; hCmPCs: human cardiac mesenchymal progenitor cells; HMGB1: high-mobility group box 1; IkBα: inhibitory κB (IκB) kinase; IL-1: interleukin-1; IL-6: interleukin-6; MyD88: myeloid differentiation factor 88; NF-kB: nuclear factor kappa-light-chain-enhancer of activated B cells; NPM: nucleophosmin; TLR4: Toll-like receptor 4; TNF-α: tumor necrosis factor-alpha; ↑: Increase; ↓: Decrease.

**Table 2:** TLR4 expression in Dox-induced cardiotoxicity: reports from in vivo studies.

Model	Protocol (Dose, Route, Duration)	Major Findings					Interpretation	Ref.
		Cardiac Function	Inflammatory Markers	Cardiac Remodeling/Fibrosis	Oxidative Stress	Apoptosis		
C57BL/6J mice	Dox (3.4 mg/kg/wk, i.p., 8 wk)	↓ %LVEF ↓ %LVFS	↑ TLR4 ↑ HMGB1 ↑ Hsp70 ↑ MCP-1 ↑ IL-13 ↑ TGF-β1	↑ Fibrosis ↑ α-SMA	n/a	n/a	Dox induced cardiac inflammation via increasing TLR4 signaling pathway, leading to cardiac dysfunction in mice.	[40]
C57BL/6J mice	Dox (15 mg/kg, i.p., single dose)	↓ %LVEF	↑ TLR4 ↑ TNF-α ↑ IL-6 ↑ NF-kB ↑ CD68 ↑ CD45	n/a	n/a	↑ Bax ↓ Bcl-2 ↑ Cyt *c* ↑ TUNEL^+^	Dox induced cardiac inflammation and apoptosis via increasing TLR4/NF-kB signaling pathway, leading to impaired cardiac function in mice.	[14]
Wistar rats	Dox (2.5 mg/kg/3 doses/wk, i.p., 2 wk)	n/a	↑ TLR4	n/a	↑ MDA	n/a	Dox induced cardiac inflammation and oxidative stress via increasing TLR4 and MDA in rats.	[23]

n/a: Data are not available; Bax: Bcl-2-associated X; Bcl-2: B-cell lymphoma-2; Cyt *c*: cytochrome c; Dox: doxorubicin; HMGB1: high-mobility group box 1; Hsp70: heat shock protein 70; i.p.: intraperitoneal injection; IL-13: interleukin 13; IL-6: interleukin-6; LVEF: left ventricular ejection fraction; LVFS: left ventricular fractional shortening; MCP-1: monocyte chemotactic protein 1; MDA: malondialdehyde; NF-kB: nuclear factor kappa-light-chain-enhancer of activated B cells; TGF-β1: tumor growth factor β1; TLR4: Toll-like receptor 4; TNF-α: tumor necrosis factor-alpha; TUNEL: terminal deoxynucleotidyl transferase mediated dUTP nick end labeling; α-SMA: α-smooth muscle actin; ↑: Increase; ↓: Decrease.

**Table 4:** The effects of Dox on systemic TLR4 expression: reports from clinical studies.

Model	Methods	Major Findings	Interpretation	Ref.
Patients with hematological malignancy who received treatment with doxorubicin (n = 25); - (Doxorubicin at 100–250 mg/m^2^, 6 months) - LVEF > 50%	- The blood was collected to determine TLR4 gene expression. - Echocardiography was used to determine cardiac function.	16 patients (64%) developed left ventricular diastolic dysfunction, associated with high gene expression of TLR4 after 6 months of Dox treatment.	The TLR4 expression may play as a marker for risk of doxorubicin-induced cardiotoxicity.	[46]
Patients with hematological malignancy who received treatment with doxorubicin (n = 25); - (Doxorubicin at 100–250 mg/m^2^, 6 wk) - LVEF > 50%	- The blood was collected to determine TLR4 gene expression. - Echocardiography was used to determine cardiac function.	There is a strong negative linear relationship between TLR4 expression and LVEF in patients after 6 weeks of Dox treatment.	Elevation of TLR4 levels were implicated in Dox-induced left ventricular dysfunction.	[47]

Dox: doxorubicin; LVEF: left ventricular ejection fraction; TLR4: Toll-like receptor 4.

## Data Availability

Not applicable.

## Abbreviations

OH	hydroxyl radical
AAV-Hsp22	adeno-associated virus-heat shock protein 22
AC16	human cardiomyocyte cell line
Ad-Hsp22	adenovirus-heat-shock protein 22
Bax	Bcl-2-associated X
Bcl-2	B-cell lymphoma-2
C34	2-acetamidopyranoside
CO	cardiac output
Cyt *c*	cytochrome c
DAMP	damage-associated molecular pattern
DIC	Dox-induced cardiotoxicity
DNA	deoxyribonucleic acid
Dox	doxorubicin
ERK	extracellular signal-regulated kinase
H_2_O_2_	hydrogen peroxide
hCmPCs	human cardiac mesenchymal progenitor cells
HMGB1	high mobility group box 1
Hsp	heat shock protein
Hsp70	heat shock protein 70
i.p.	intraperitoneal injection
IKK	inhibitory κB kinase
IL-1β	interleukin-1 beta
IL-6	interleukin-6
JNK	c-Jun N-terminal kinase
LPS	lipopolysaccharide
LVESPVR	left ventricular end-systolic pressure-volume relation
MAPK	mitogen-activated protein kinase
MCP-1	monocyte chemotactic protein 1
MD2	myeloid differentiation factor 2
MDA	malondialdehyde
MyD88	myeloid differentiation factor 88
NPM	nucleophosmin
O_2_^−^	superoxide anion
PAMP	pathogen-associated molecular pattern
ROS	reactive oxygen species
SV	stroke volume
TGF-β1	tumor growth factor β1
TLR4	Toll-like receptor 4
TNF-α	tumor necrosis factor-alpha
TUNEL	Terminal deoxynucleotidyl transferase dUTP nick end labeling
VA	vanillic acid
α-SMA	α-smooth muscle actin
